# Identification of a 5-Hydroxymethylation Signature in Circulating Cell-Free DNA for the Noninvasive Detection of Colorectal Cancer

**DOI:** 10.1155/2022/3798741

**Published:** 2022-10-12

**Authors:** Hongwei Liu, Tao Tang, Huixian Zhang, Weiren Ting, Peng Zhou, Ying Luo, Huaxing Qi, Yanmei Liu, Yongxin Liu, Meifang Zhou, Weiguo Yin, Jinduan Lin

**Affiliations:** ^1^Department of Laboratory Medicine, The Sixth Affiliated Hospital of Guangzhou Medical University, Qingyuan People's Hospital, Guangdong Qingyuan, China; ^2^Department of Molecular Diagnostics, Sun Yat-sen University Cancer Center, Guangdong Guangzhou, China

## Abstract

**Background:**

As a crucial epigenetic modification, DNA 5-hydroxymethylcytosine (5-hmC) plays a key role during colorectal cancer (CRC) carcinogenesis. Nevertheless, the levels of 5-hmC-related genes in the circulating DNA of CRC remain largely unknown.

**Methods and Results:**

The GSE81314 dataset from the Gene Expression Omnibus (GEO), which was generated by chemical marking-based low-input shotgun sequencing to detect 5-hmC in circulating cell-free DNA (cfDNA) was used in the present study. The GSE81314 dataset includes data for 8 plasma samples from healthy individuals and 4 plasma samples from CRC patients. The difference in the 5-hmC levels in cfDNA between the CRC group and healthy individuals was analyzed by the differentially expressed genes (DEG) package. Weighted gene coexpression network analysis (WGCNA) was conducted to analyze gene coexpression modules associated with sample characteristics. DEG analysis identified 19 upregulated and 9 downregulated 5-hmC-related genes. WGCNA showed that the pink, purple, and brown modules, which contain 531 genes in total, were significantly correlated with CRC (0.66, 0.61, and -0.59, respectively). We used gene set enrichment analysis (GSEA) software to compare 5-hmC-related genes and pathways between CRC patients and healthy controls. We further performed a protein–protein interaction (PPI) analysis and identified 4 nodes (LCN2, LRG1, S100P, and TACSTD2) that played key roles in the network, and we analyzed the expression of these nodes S100P in the GEPIA database. Consistent with the 5-hmC levels in CRC patient plasma, our external validation results from the GEPIA and UALCAN databases showed that LCN2, LRG1, S100P, and TACSTD2 were highly expressed in CRC tissue compared with controls. The DNA promoter methylation levels of LCN2, LRG1, and S100P were lower in CRC tissue than in normal control tissue.

**Conclusion:**

The present findings suggest that abnormality in cell-free DNA hydroxylation in plasma may be associated with CRC. In addition, the 5-hmC levels of LCN2, LRG1, S100P, and TACSTD2 in circulating cfDNA may be used as potential noninvasive markers for CRC.

## 1. Introduction

Colorectal cancer (CRC), which involves neoplastic transformation in normal intestinal epithelial cells due to the accumulation of anomalous genetic and epigenetic alterations, is one of the most frequent malignant gastrointestinal neoplasms worldwide. The diagnosis of CRC is showing an upward trend in China [1–5]. Colonoscopy, as the gold standard method for detecting colorectal cancer, is widely used for screening and has an obvious effect on the early diagnosis, prevention, and treatment of CRC. However, endoscopic screening requires a specific time for preparation, bowel preparation, and laxatives to ensure visibility of the intestinal tract, and it is invasive and uncomfortable, thereby dissuading participation. Therefore, the compliance rate of colonoscopy is still low. Recently, the use of easily accessible sample types, such as stool and blood, for CRC noninvasive screening has gradually been applied in the clinic due to their advantages compared to colonoscopy. Blood-based liquid biopsy has been utilized in the clinic for CRC noninvasive screening because it is noninvasive and rapid, and it is more acceptable than stool-based assays [6]. In recent studies, circulating cell-free DNA (cfDNA) has become the predominant tool for liquid biopsies to understand the mutational landscape of cancer. Cancer cells shed naked DNA molecules into the circulation, and these molecules are called circulating tumor DNA (ctDNA). ctDNA has been used for clinical diagnosis and prognosis prediction. Few but promising data are available about the use of liquid biopsy for the early diagnosis of CRC, and the main limitation is sensitivity due to low concentrations of ctDNA in this setting. In terms of the prediction of the response to chemoradiation, only inconclusive data are available about the utility of a pretreatment liquid biopsy, whereas some studies report a positive correlation with dynamic (pre/posttreatment) monitoring. The presence of minimal residual disease by ctDNA is consistently associated with a poor prognosis across studies [7]. Laboratory tests of DNA-related epigenetic changes in cfDNA can be performed from plasma or serum fractions, which are primarily derived from tumor cells. Epigenetic changes are important causes of CRC, and abnormal DNA methylation and 5-hmC modification impair cancer development and progression [8–11]. 5-hmC is generated from 5-mC by the Tet protein family. 5-hmC modifications are ubiquitous in the DNA of embryonic stem cells and many other issues, and they are implicated in many human diseases, including CRCs [10–12]. Li et al. found robust cancer-associated 5-hmC signatures in cfDNA that were characteristic of specific cancer types. 5-hmC-based biomarkers of circulating cfDNA are highly predictive of colorectal and gastric cancer prognosis and are superior to conventional biomarkers and comparable to 5-hmC biomarkers from tissue biopsies [13]. Nonetheless, no previous studies have investigated the potential for CRC detection based on the presence of colorectal-related 5-hmC genes in circulating cfDNA, a concept that may help to develop noninvasive screening tools in CRC detection.

In the present study, microarray data (GSE81314) were used to compare the 5-hmC levels of hub genes between healthy controls and CRC patients. First, we downloaded the data (GSE81314) from the GEO database and analyzed the differences in the 5-hmC level of cfDNA in plasma between the healthy and CRC groups by the DEG package. We then performed weighted gene coexpression network analysis (WGCNA) to identify hub genes at the 5-hmC level that are closely related to CRC. In addition, protein–protein interaction (PPI) studies were conducted to determine the interaction network of genes that showed critical expression. Finally, we verified the expression level, stage characteristics, and survival time of the 5-hmC-related genes between the CRC and healthy control groups in the Gene Expression Profiling Interactive Analysis (GEPIA) database. The present study aimed to identify 5-hmC-related genes and pathways that are highly associated with CRC in plasma and to elucidate the potential mechanisms.

## 2. Materials and Methods

### 2.1. Data

The GSE81314 dataset was downloaded from the Gene Expression Omnibus (GEO) database (https://www.ncbi.nlm.nih.gov/geo/query/acc.cgi?acc=GSE81314). This dataset contains sequenced cfDNA with 5-hmC data from 49 patients with seven different cancer types, including 8 plasma samples from healthy individuals and 4 plasma samples from CRC patients, and it also contains distinct features that can be used for monitoring disease status and progression. The dataset was based on the GPL18573 Illumina NextSeq 500 (Homo sapiens) platform.

### 2.2. DEG Analysis

We analyzed the DEG to evaluate the different 5-hmC levels of cfDNA in plasma between healthy individuals and CRC patients by the limma package [14]. The parameter settings were set according to a previous study [15].

### 2.3. WGCNA

We used the WGCNA package [16] to determine the coexpression modules and the interconnectedness between each module, and the coexpressed genes in the control and CRC patients according to a previously published method [15].

### 2.4. GSEA

We downloaded the hallmark gene set and gene symbols from the GSEA website to obtain 5-hmC-associated genes in CRC.

### 2.5. GO and KEGG Pathway Enrichment Analyses

We used Gene Ontology (GO) [17] to annotate the GO function of 5-hmC-related genes in cfDNA, and we performed Kyoto Encyclopedia of Genes and Genomes (KEGG) [18] pathway analysis to determine the 5-hmC-related signaling pathways. GO and KEGG pathway analyses were performed to identify the related functions of 5-hmC-related genes, and they were based on the DEG and WGCNA results, which provided 5-hmC-related genes significantly associated with CRC. The threshold for statistical significance was set at *p* < 0.05. The detailed operation procedure was performed as previously reported [15].

### 2.6. PPI Network Analysis

GeneMANIA software [19] was used to assess gene interactions and to predict gene function. The 5-hmC-related genes in the cfDNA of CRC patients were identified using the GeneMANIA plugin in Cytoscape 3.8.0. Network analysis was performed according to methods described in a previous study [15]. Genes with degree values of three or greater were considered hub genes and used for further analysis.

### 2.7. Analysis of the RNA Expression Level and DNA Promoter Methylation of 5-hmC-Related Hub Genes in CRC Tissue in Public Databases

We analyzed the mRNA levels analyzing the DNA promoter methylation levels of the 5-hmC-related target genes linked to CRC via the GEPIA database [20] (including 275 CRC tissues and 349 normal samples from the TCGA and the GTEx projects), and the UALCAN database [21] (including 41 normal tissues and 286 primary CRC tissues for mRNA expression analysis, 37 normal tissues and 313 primary CRC tissues for prompter methylation level analysis, and 279 CRC cases for survival analysis).

### 2.8. Analysis of the Correlations of 5-hmC-Related Hub Genes with Clinical Characteristics in Public CRC Datasets

We analyzed the relationship between CRC stage, overall survival, and the mRNA expression levels of the 5-hmC-related target genes linked to CRC in the GEPIA database and the UALCAN database.

## 3. Results

### 3.1. DEG Analysis

**Figure 1:**
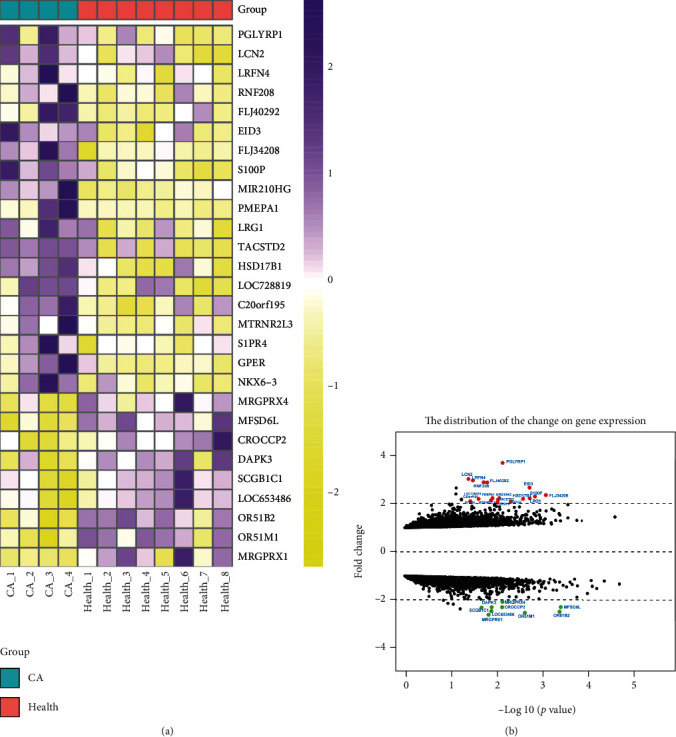
Heatmap clustering and volcano plot of 5-hmC-related DEG in the plasma of CRC patients and healthy individuals. (a) Heatmap clustering of the DEG. The red bars represent the CRC group, and the green bars represent the healthy group. (b) Volcano plot of the DEG. The red nodes represent upregulated DEG, and the green nodes represent downregulated DEG.

The 5-hmC levels of the hub genes in plasma between healthy controls and CRC patients were analyzed by the DEG package.  Figure 1 shows that 28 genes, including 19 upregulated genes and 9 downregulated genes, were differentially expressed. The average logFC values of the upregulated and downregulated genes were 1.35 and -1.25, respectively. The genes with logFC >1 or logFC <-1 are shown in Table 1.

### 3.2. WGCNA

**Figure 2:**
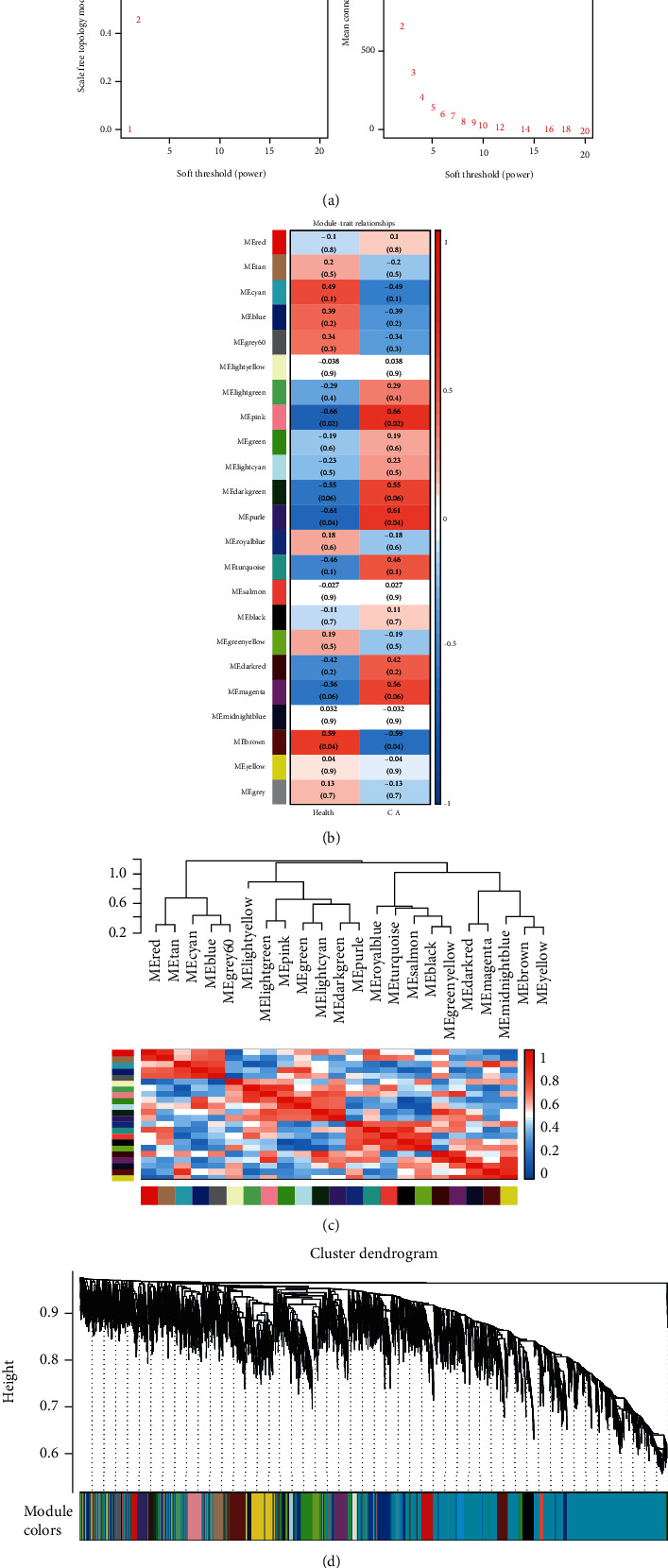
Identification of key modules associated with CRC. (a) Network topology of different soft-thresholding powers of the CRC coexpression network. (b–c) Heatmap of the correlation between module eigengenes and the two groups of plasma. (d) 5-hmC gene clustering module of the CRC coexpression network.

With a soft threshold of 4, all genes were grouped into 23 modules by cluster analysis, and the correlations of the 5-hmC level genes with phenotype were analyzed. As shown in Figure 2, the pink, purple, and brown modules were significantly correlated with CRC with values of 0.66, 0.61, and -0.59, respectively. These three modules contained 531 genes in total (pink, 147; purple, 84; and brown, 300). The detailed results are shown in Supplement table 1.

### 3.3. GSEA, GO, and KEGG Analyses

**Figure 3:**
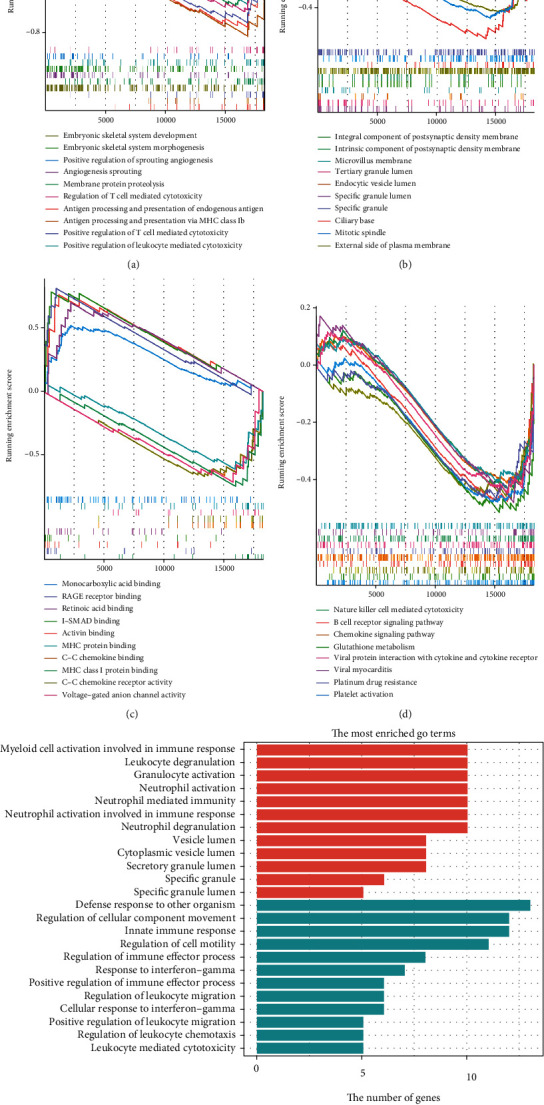
Potential functions of the 5-hmC-related genes in plasma according to GSEA. Plasma 5-hmC-related genes of CRC were significantly enriched in the listed BPs (a), CCs (b), MFs (c), and KEGG pathways (d).

We performed GSEA software to extract the 5-hmC-associated genes and pathways between CRC patient and healthy control plasma. GSEA and GO results of the microarray data (GSE81314) showed that the upregulated 5-hmC-related genes were highly enriched in angiogenesis sprouting, specific granule lumen, antigen processing, embryonic skeletal system development, and positive regulation of T-cell-mediated cytotoxicity. The 5-hmC-associated genes upregulated in CRC patients compared to healthy controls were enriched in pathways related to immune functions, platelet activation, and platinum drug resistance (Figures 3(a)–3(d)). The other related pathways are shown in Table 2.

### 3.4. GO and KEGG Pathway Enrichment Analyses

GO, Reactome, and KEGG pathway analyses were utilized to determine the upregulated 5-hmC genes, downregulated 5-hmC genes, and 5-hmC genes significantly correlated with CRC (pink, purple, and brown modules). The GO results indicated that the upregulated 5-hmC-related genes were highly enriched in leukocyte activation involved in the immune response and secretory activity process. The downregulated DEG was mainly associated with the cellular chemotaxis process (Table 2).

In CRC, the genes in the 5-hmC-related modules were enriched in platelet activation, the B-cell receptor signaling pathway, the chemokine signaling pathway, cytokine–cytokine receptor interaction, and the cell metabolism-related pathway. The KEGG and Reactome pathway results indicated the involvement of the upregulated molecules in the B-cell signaling pathway and in diseases of the immune system (Figure 3(e) and Table 3).

### 3.5. PPI Network Analysis

**Figure 4:**
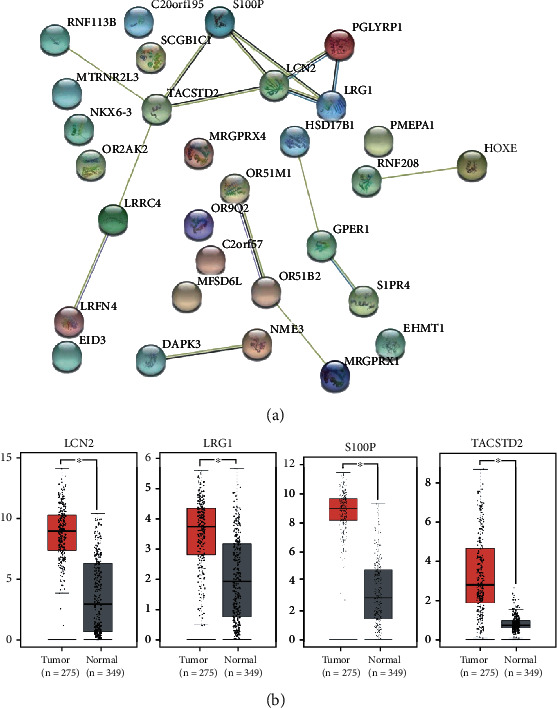
PPI network analysis and expression of plasma 5-hmC hub genes in tissue. (a) PPI network analysis of the DEG. (b) Expression levels in tissue of the plasma 5-hmC hub genes between CRC and healthy controls in the GEPIA database.

PPI network analysis was performed to investigate the 28 genes that were both DEG and in the highly correlated modules. Based on previously reported criteria [15], we obtained a total of four genes, all of which were upregulated. As shown in Figure 4(a), the four molecules with the highest degree value in the network were LCN2 (logFC =1.88), S100P (logFC =1.24), TACSTD2 (logFC =1.16), and TACSTD2 (logFC =1.15), and these genes significantly impacted the network.

### 3.6. Exploration of the Expression and DNA Promoter Methylation Levels of Critical Genes

**Figure 5:**
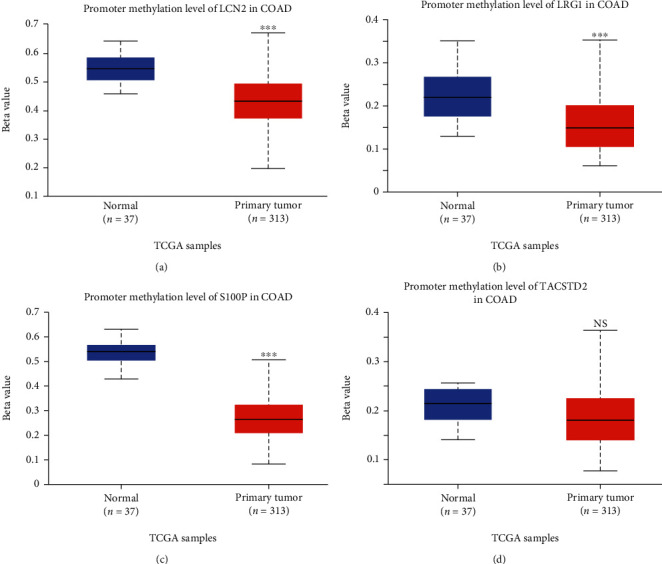
External validation of DNA promoter methylation of the four 5-hmC hub genes in CRC tissue in UALCAN database. DNA promoter methylation levels in tissue of LCN2 (a), LRG1 (b), S100P (c), and TACSTD2 (d) between CRC and healthy controls in the UALCAN database.

The four genes that had a significant influence on the PPI network were used for further analysis. We used the GEPIA database to analyze the expression difference between CRC and normal tissues. Consistent with our results, the four genes were all upregulated in CRC tissues (Figure 4(b)). 5-Methylcytosine (5mC) in DNA can be iteratively oxidized by Tet proteins to generate 5-hmC, which can be further processed by thymine-DNA glycosylase (TDG) followed by base excision repair or by replication-dependent dilution leading to DNA demethylation. In summary, Tet proteins suppress gene methylation by increasing the 5-hmC level. We performed external validation of this mechanism by exploring the DNA promoter demethylation level of the hub genes in the public database UALCAN. As expected, the DNA promoter demethylation levels of LCN2, LRG1, and S100P were significantly lower in CRC tissue than in the normal tissues (Figure 5). Because the DNA promoter demethylation level can often affect the RNA level, lower DNA promoter methylation level can often lead to a higher RNA expression level, which is consistent with the results shown in Figure 4(b).

### 3.7. Analysis of the Correlations of 5-hmC-Related Hub Genes with Clinical Characteristics in Public CRC Datasets

**Figure 6:**
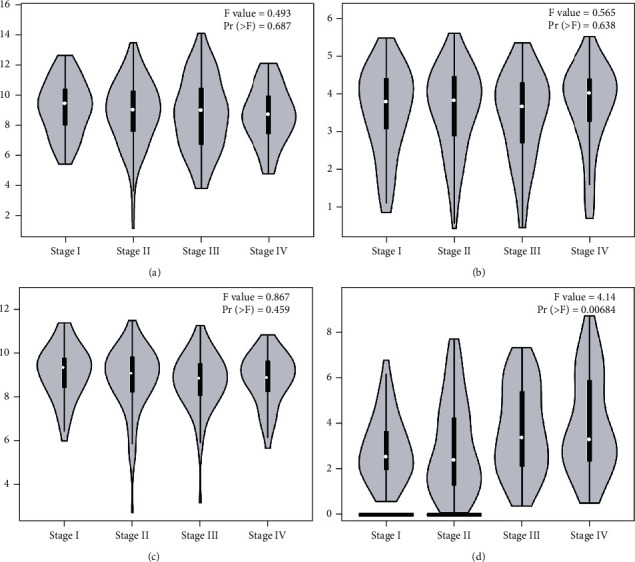
Analysis of the correlation between the 5-hmC-related hub genes and CRC stage in GEPIA database. The correlations between LCN2 (a), LRG1 (b), S100P (c), and TACSTD2 (d) and CRC stage in the GEPIA database.

**Figure 7:**
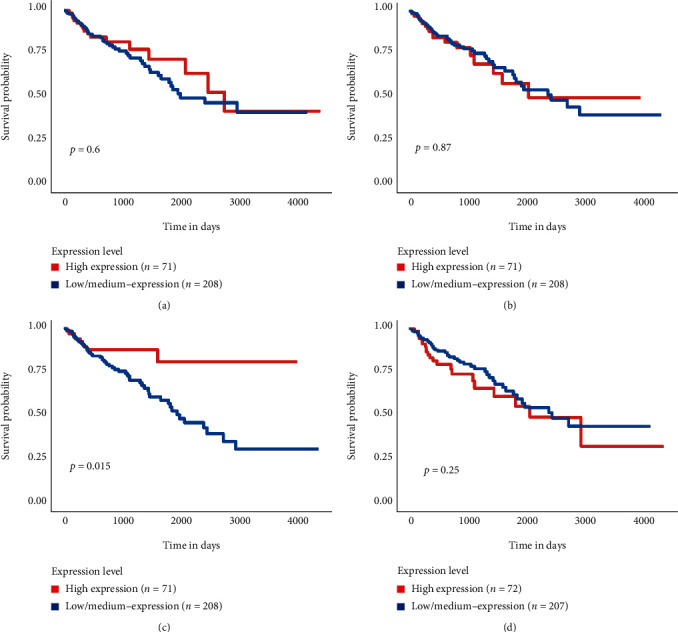
Analysis of the correlation between the 5-hmC-related hub genes and CRC patients' overall survival time in the UALCAN database The correlation between LCN2 (a), LRG1 (b), S100P (c), and TACSTD2 (d) and CRC patients' overall survival time in the UALCAN database.

The four genes that had a significant influence on the PPI network were used for clinical correlation analysis. We used the GEPIA database to analyze the mRNA expression levels of the 5-hmC related hub genes at different CRC stages. We also explored the relationship between the overall survival time and the mRNA levels of the four genes in the UALCAN database. We found that TACSTD2 was associated with the stage of CRC (Figure 6), and S100P was associated with the overall survival of CRC patients. A high expression level of S100P was a predictor of a poor prognosis in CRC patients (Figure 7).

## 4. Discussion

Recent studies have reported that 5-hmC may be associated with human cancer [22, 23]. In the present study, the 5-hmC level of the hub genes in CRC patients was different from that in healthy patients. Our study found 19 upregulated genes and 9 downregulated 5-hmC-related hub genes in plasma between healthy controls and CRC patients. Next, we set the criteria of degree more than or equal to three to screen hub genes as biomarkers of circulating cfDNA in cancer by PPI analysis The present findings suggested that the 5-hmC levels of LCN2, LRG1, S100P, and TACSTD2 in circulating cfDNA may be used as potential noninvasive marker genes for CRC, resulting in several notable advantages. First, in contrast to previous genome-wide analyses of hydroxylation, we used sequencing and bioinformatics to analyze the differential level of hydroxylation-related genes between CRC patients and healthy controls. Second, we analyzed genes with different levels of hydroxylation modification of cfDNA in plasma to offer a new strategic method for noninvasive screening of CRC.

In humans, DNA methylation is an important epigenetic modification that is closely related to tumor development and progression. DNA methylation includes various patterns, such as 5-mC and 5-hmC. 5-mC is converted by oxidative demethylation by the ten-eleven translocation enzyme family (TET1, TET2, and TET3) or by passive demethylation of copies [24]. It is reported that 5-methylcytosine (5mC) in DNA can be iteratively oxidized by a family of proteins known as Tet proteins to generate 5-hydroxymethylcytosine (5hmC), which can be further processed by thymine-DNA glycosylase (TDG) followed by base excision repair or replication-dependent dilution leading to DNA demethylation. In summary, Tet proteins downregulated gene methylation by upregulating the 5-hmc level [21]. In addition, 5-hmC levels are low in many cancers, including CRC [25, 26]. Li etal found that the correlation of 5-hmC changes in cancer between the discovery and validation datasets was higher in plasma cfDNA (cancer patients vs. healthy individuals) than in tissue gDNA (tumors vs. adjacent tissues), especially for 5-hmC in gene bodies. The predicted cancer probability based on the 5-hmC classifier from plasma cfDNA showed a significant trend associated with clinical stage. After surgery, patients had predicted scores undistinguishable from those of healthy individuals. However, the hub 5-hmC-related genes for CRC remain largely unknown, and whether 5-hmC-related genes can be used as CRC diagnostic biomarkers needs to be verified in future studies [13]. In the present study, we identified aberrant changes in the 5-hmC levels of 28 genes in the plasma of CRC individuals. WGCNA, GSEA, and GO analysis demonstrated that the 5-hmC levels of some hub genes in the plasma of CRC patients were significantly different than those in the plasma of healthy controls, and these hubs were mainly enriched in the immune response, angiogenesis, drug resistance, and other related signaling pathways. Cell motility is a complex, multistep, and multicomponent process intrinsic to progression and metastasis. Motility is dependent on the activities of integrin receptors and Rho family GTPases, resulting in the remodeling of the actin cytoskeleton and the formation of various motile actin-based protrusions [27]. In our study, GO and KEGG pathway enrichment analyses showed that the 5-hmC level of motility pathway-related genes was downregulated in CRC patients. The lower 5-hmC level of motility pathway-related genes was related to increase mRNA expression of motility pathway-related genes. This may play an important role in CRC progression and metastasis. GO and KEGG pathway enrichment analyses showed that the B-cell receptor signaling pathway related gens was in a higher 5-hmC level in CRC patients' plasma but T-cell immunity was not. This phenomenon may be related to the type of sample we studied. In this study, we used plasma to detect 5-hmC in circulating cfDNA. B-cell immunity is humoral immunity, which mainly depends on antibodies and receptors. Its receptors and antibodies can exist in plasma. T-cell immunity is a type of cellular immunity and mainly plays a role in tissues. T-cells in the blood mainly exist in the monolayer. Because we used plasma samples, no differences were detected in the T-cell immunity pathway. Our study found elevated levels of 5-hmC in the B-cell receptor pathway in CRC patients, suggesting that the B-cell receptor pathway is inhibited in CRC patients.

The PPI network analysis and further analyses in the GEPIA and UALCAN databases suggested that LCN2, LRG1, S100P, and TACSTD2 are both expressed and methylated in the plasma of CRC patients. 5-hmC is used as a marker of DNA demethylation. Examples of aberrant methylation levels in the genome include hypermethylation of tumor suppressor genes and hypermethylation of oncogenes. Although PGLYRP1 is the highest difference expression gene in the DEGs analysis list, it shows that PGLYRP1 is only two related genes in PPI analysis. So, we did not verified PGLYRP1 in the GEPIA database or ULCAN database.

LCN2, also known as neutrophil gelatinase-associated lipocalin (NGAL), was first discovered as a protein related to neutrophil gelatinase. Recent studies have shown that LCN2 is mainly involved in cellular immunity. LCN2 expression starts in the fetal stages but is fairly low in healthy adult humans. LCN2 has been reported to play a key role in the development and progression of several tumors, such as breast cancer [28], thyroid cancer, CRC, non-small-cell lung cancer, hepatocellular carcinoma, and leukemia [27, 29]. In most cancers, LCN2 is upregulated, but the underlying mechanism remains largely unknown. The present study demonstrated that LCN2 was upregulated in CRC tissue and that the 5-hmC level of LCN2 in CRC patient plasma was also upregulated. We speculated that in CRC patients, the 5-hmC level of LCN2 is increased, leading to upregulated LCN2 demethylation, thereby promoting its expression. LRG1 is a highly conserved member of the leucine-rich repetitive sequence family, which was first identified in human serum in 1977 [30]. LRG1 is a secreted glycoprotein that mediates the interaction between proteins and has been studied as a tumor-promoting factor that participates in signal transduction, cell proliferation, migration, invasion, adhesion, survival, and apoptosis [31–33]. Previous studies have identified LRG1 as a new proangiogenic gene that enhances cancer growth and diabetic retinopathy. Zhang found that overexpression of LRG1 significantly enhances the migration and tube formation capabilities of HUVECs [34]. Similar to LCN2, the present study showed that the 5-hmC and mRNA levels of LRG1 were upregulated in CRC patients compared to healthy individuals. LRG1 was enriched in the angiogenesis-related pathway. Abnormal levels of TACSTD2 are associated with the progression of many tumors. The present study demonstrated that TACSTD2 was upregulated in CRC tissue and CRC patient plasma compared to plasma from healthy individuals, and the mRNA level of TACSTD2 is positive associated with CRC stage. Similar to our study, Katzendorn et al. reported that the DNA methylation of TACSTD2 loci is related to clinically aggressive renal cell cancers [35]. S100P is S100 calcium binding protein P, and the protein encoded by this gene is a member of the S100 family of proteins containing 2 EF-hand calcium-binding motifs. S100 proteins are localized in the cytoplasm and/or nucleus of a wide range of cells and are involved in the regulation of a number of cellular processes such as cell cycle progression and differentiation. Recent studies have reported that S100P is a new target gene of MACC1 that drives colorectal cancer metastasis and serves as a prognostic biomarker [36]. Consistent with this, we found that the 5-hmC level of S100P in CRC patients' plasm was significant higher than that in the plasma of healthy individuals. The mRNA expression level was upregulated in CRC tissue, which was associated with a poor prognosis in CRC patients. It is reported that DNA hydroxymethylation increases the susceptibility to reactivation of methylated P16 alleles in cancer cells, and 5-hmc may play an important role in gene transcription [37]. This may partly explain our research that LCN2, LRG1, S100P, and TACSTD2 DNA hydroxymethylation levels were positively correlated with their mRNA levels. In addition, the 5-hmC levels ofLCN2, LRG1, S100P, and TACSTD2 in circulating cfDNA may be used as potential noninvasive markers for CRC. Although we found that abnormalities in cell-free DNA hydroxylation in plasma may be associated with an abnormal immune response to CRC. However, this study only used 4 CRC samples from the GEO, so the number of samples is limited, and more samples are needed for validation. On the other hand, due to the level of 5-hmC in the blood is very low, quantitative and high-resolution analysis of active DNA demethylation activity remains challenging. We have not verified the 5-hmC level of the hub genes in clinical samples; we are looking forward to advances in the new technology. In addition, the underline mechanism on how the LCN2, LRG1, S100P, and TACSTD2 function in CRC occurrence, development, and outcome needs to be explored in the further researches.

## 5. Conclusion

The abnormal expression of some 5-hmC-related genes in the plasma of patients with CRC may influence the 5-hmC expression level through the methylation level of related genes in CRC. The 5-hmC level of some genes in plasma may act as biomarker for CRC.

## Figures and Tables

**Table 1:** Different 5-hmC levels of hub genes with logFC>1.

	logFC	*t*	*P* value
PGLYRP1	2.55	2.77	0.012
LCN2	1.88	3.20	0.008
LRFN4	1.60	2.28	0.041
RNF208	1.57	2.40	0.033
FLJ40292	1.53	2.70	0.019
EID3	1.53	2.79	0.016
FLJ34208	1.41	3.94	0.002
S100P	1.24	4.40	0.001
MIR210HG	1.20	4.09	0.001
PMEPA1	1.16	3.11	0.009
LRG1	1.16	2.93	0.013
TACSTD2	1.15	3.94	0.002
HSD17B1	1.13	3.76	0.003
LOC728819	1.13	2.56	0.025
C20orf195	1.11	3.08	0.009
MTRNR2L3	1.10	2.89	0.013
S1PR4	1.07	2.33	0.038
GPER	1.06	3.42	0.005
NKX6-3	1.03	3.07	0.009
MRGPRX4	-1.06	-3.20	0.008
MFSD6L	-1.20	-4.83	0.0004
CROCCP2	-1.20	-3.19	0.008
DAPK3	-1.20	-2.91	0.0132
SCGB1C1	-1.21	-2.64	0.022
LOC653486	-1.30	-2.90	0.013
OR51B2	-1.31	-4.79	0.0004
OR51M1	-1.34	-3.81	0.002
MRGPRX1	-1.39	-2.83	0.015

**Table 2:** GO analysis of the DEG and genes in the highly correlated modules.

GO ID	Description	*P* value	No. genes
Upregulated genes			
GO:0035580	Specific granule lumen	2.83E-06	5
GO:0043312	Neutrophil degranulation	8.04E-06	10
GO:0002283	Neutrophil activation involved in immune response	8.44E-06	10
GO:0002446	Neutrophil-mediated immunity	1.00E-05	10
GO:0042119	Neutrophil activation	1.00E-05	10
GO:0036230	Granulocyte activation	1.12E-05	10
GO:0043299	Leukocyte degranulation	1.81E-05	10
GO:0042581	Specific granule	1.97E-05	6
GO:0034774	Secretory granule lumen	2.00E-05	8
GO:0060205	Cytoplasmic vesicle lumen	2.16E-05	8
GO:0002275	Myeloid cell activation involved in immune response	2.18E-05	10
GO:0031983	Vesicle lumen	2.25E-05	8
GO:0002444	Myeloid leukocyte-mediated immunity	2.41E-05	10
GO:0002274	Myeloid leukocyte activation	9.22E-05	10
GO:0030141	Secretory granule	1.58E-04	11
GO:0002366	Leukocyte activation involved in immune response	1.76E-04	10
GO:0002263	Cell activation involved in immune response	1.85E-04	10
GO:0031960	Response to corticosteroid	3.83E-04	5
GO:0045055	Regulated exocytosis	4.58E-04	10
GO:0099503	Secretory vesicle	6.86E-04	11
GO:0002443	Leukocyte-mediated immunity	1.13E-03	10
Downregulated genes			
GO:0034341	Response to interferon-gamma	2.54E-06	7
GO:0071346	Cellular response to interferon-gamma	1.92E-05	6
GO:0001909	Leukocyte-mediated cytotoxicity	2.04E-05	5
GO:0045087	Innate immune response	2.57E-05	12
GO:0098542	Defense response to other organisms	3.88E-05	13
GO:0002688	Regulation of leukocyte chemotaxis	4.38E-05	5
GO:0051270	Regulation of cellular component movement	4.70E-05	12
GO:0002685	Regulation of leukocyte migration	4.86E-05	6
GO:0002687	Positive regulation of leukocyte migration	7.21E-05	5
GO:0002699	Positive regulation of immune effector process	7.40E-05	6
GO:0002697	Regulation of immune effector process	9.47E-05	8
GO:2000145	Regulation of cell motility	1.01E-04	11
GO:0040012	Regulation of locomotion	1.46E-04	11
GO:0002703	Regulation of leukocyte-mediated immunity	4.34E-04	5
GO:0050920	Regulation of chemotaxis	6.42E-04	5
GO:0030595	Leukocyte chemotaxis	7.56E-04	5
GO:0004930	G protein-coupled receptor activity	9.04E-04	9
GO:0032103	Positive regulation of response to external stimulus	9.04E-04	7
GO:0030335	Positive regulation of cell migration	1.03E-03	7
GO:2000147	Positive regulation of cell motility	1.31E-03	7
GO:0040017	Positive regulation of locomotion	1.53E-03	7

Notes. GO, Gene Ontology; DEG, differentially expressed genes.

**Table 3:** KEGG and Reactome pathway enrichment analyses of the DEG and genes in the highly correlated modules.

ID	Description	*P* value	NES_abs
hsa04611	Platelet activation	1.96E-03	1.82
hsa00480	Glutathione metabolism	2.02E-03	1.72
hsa05414	Dilated cardiomyopathy	2.01E-03	1.70
hsa04662	B-cell receptor signaling pathway	2.00E-03	1.69
hsa04062	Chemokine signaling pathway	2.10E-03	1.65
hsa01524	Platinum drug resistance	5.93E-03	1.65
hsa04650	Natural killer cell-mediated cytotoxicity	2.00E-03	1.63
hsa00410	Beta-alanine metabolism	3.75E-02	1.57
hsa00670	One carbon pool by folate	2.94E-02	1.51
hsa04060	Cytokine–cytokine receptor interaction	3.96E-03	1.51
hsa04550	Signaling pathways regulating pluripotency of stem cells	8.08E-03	1.49
hsa00515	Mannose type O-glycan biosynthesis	4.70E-02	1.48
hsa02010	ABC transporters	3.43E-02	1.47

Abbreviations. KEGG, Kyoto Encyclopedia of Genes and Genomes; DEG, differentially expressed genes.

## Data Availability

All data generated or analyzed during this study are included in this published article.

## Abbreviations

5-hmC: 5-hydroxymethylcytosine
CRC: Colorectal cancer
GEO: Gene expression omnibus
DEG: Differentially expressed genes
WGCNA: Weighted gene coexpression network analysis
GO: Gene ontology
PPI: Protein–protein interaction
cfDNA: Circulating cell-free DNA.

## Supplementary Materials

Supplementary Materials The correlations between the 5-hmC level with phenotype by WGCNA.
