# Supplementary material for: Identification of a 5-Hydroxymethylation Signature in Circulating Cell-Free DNA for the Noninvasive Detection of Colorectal Cancer
Source: J Oncol. 2022 Oct 12;2022:3798741. doi: 10.1155/2022/3798741 (PMC9581595; doi:10.1155/2022/3798741)
Supplement: Supplementary Materials — The correlations between the 5-hmC level with phenotype by WGCNA. [file 3798741.f1.docx]

Supplement table 1 The correlations of the 5-hmC level genes with phenotype were analyzed by WGCNA

| pink model(p=0.02) | |  | purple model(p=0.04) |  | brown model(p=0.02) | | | |
| --- | --- | --- | --- | --- | --- | --- | --- | --- |
| C20orf195 | FKBP1A |  | LOC100288123 |  | PGLYRP1 | ITPRIP | PAPD7 | MPZL3 |
| LOC100287042 | C14orf80 |  | CROCCP2 |  | LCN2 | CRIP1 | B3GALT6 | PROB1 |
| CCL3 | PLCG1 |  | TMEM52 |  | LRRC4 | WNT11 | TP53I11 | MTRNR2L6 |
| SPHAR | SUN2 |  | PDXDC1 |  | EID3 | MMP11 | IGFBP6 | THAP1 |
| TMEM160 | CTBP1-AS1 |  | HLA-A |  | GPR182 | POMT1 | HRASLS2 | ZKSCAN4 |
| C5AR2 | PDE12 |  | SLC52A1 |  | CEBPE | DGKZ | ZNF256 | XKR8 |
| NCR3 | CDH24 |  | SAP25 |  | LRG1 | CD1E | PHGR1 | GRAMD1A |
| LBX2-AS1 | ST6GALNAC1 |  | HCG4B |  | UBL4B | FXYD1 | ASCL2 | MICAL1 |
| C1orf229 | ALDH4A1 |  | CAMP |  | MGC16025 | IRF9 | LOC729020 | TRPV3 |
| LOC494558 | SERPINF1 |  | FAM132A |  | SPATA25 | CFD | DUSP13 | SCO2 |
| SAT2 | MICB |  | GSTM5 |  | AP5B1 | BCKDHA | RAB7L1 | ATP2A2 |
| GPRIN2 | MXRA7 |  | GPR52 |  | S100P | SLC2A4RG | HLA-H | FAM47E-STBD1 |
| BCKDK | DKFZP434A062 |  | CDRT15L2 |  | FOLR3 | BRI3 | CORO2A | PRSS22 |
| C14orf142 | SCGB2B2 |  | DUSP22 |  | GAMT | FLJ20021 | RCVRN | LINC00854 |
| CYBA | LOC339442 |  | CEND1 |  | GPR84 | TCF23 | TAGAP | LOC440335 |
| RSC1A1 | ACSL1 |  | KRTAP10-1 |  | TACSTD2 | GCSHP3 | SNAPC2 | ZNF835 |
| KAAG1 | FAM45B |  | SLC7A5P2 |  | ODF3 | PXN | PCSK6 | ARL2 |
| RNF138P1 | CCNO |  | ATHL1 |  | MSRB1 | OR4N4 | SYTL1 | GYS2 |
| TLR9 | DCDC2B |  | LOC100131096 |  | LTF | MXD3 | CEACAM6 | FLJ14186 |
| LOC100499489 | TMEM88B |  | FIS1 |  | NSUN5 | STOML2 | CEACAM3 | MTRNR2L5 |
| GPER | CHRNE |  | C19orf35 |  | SPDYC | FSTL3 | TUSC2 | GHRH |
| FLJ34208 | FAM45A |  | KBTBD6 |  | POU5F2 | LPCAT1 | HTR1B | TMEM92 |
| HYAL2 | IL10RA |  | MTFP1 |  | MLNR | TPI1 | CYP17A1 | LOC283214 |
| RARRES2 | CBX2 |  | PANX2 |  | OXLD1 | FITM2 | TEDDM1 | FOXI2 |
| HCG9 | PAQR7 |  | HIGD1B |  | METRNL | LDOC1L | KRTAP4-11 | LINC00563 |
| TMEM139 | LOR |  | RPL18 |  | CLDN3 | LOC100862671 | ICT1 | HLA-C |
| C19orf26 | SMCR7 |  | RHBDD3 |  | CEACAM8 | ZMYND10 | FLJ10038 | RPUSD2 |
| C8G | HERPUD1 |  | HSD17B8 |  | LCAT | HUS1B | ANXA11 | ZMAT2 |
| C21orf67 | KIAA0930 |  | AQP7 |  | EPN2-IT1 | LOC150381 | LOC100129931 | TUBB6 |
| VWA1 | CGB5 |  | CROCC |  | C1orf35 | CHCHD1 | LOC100653515 | ACP2 |
| ARHGAP1 | MCF2L |  | LOC100288778 |  | EPN2-AS1 | BATF2 | QPRT | NXN |
| RPS18 | ACOX1 |  | SLC25A52 |  | CSF3 | RAMP3 | CNTFR | SLC18A3 |
| SERPINF2 | TMEM203 |  | SECTM1 |  | RHBDL1 | SPIC | TCN1 | GMNN |
| MYC | RASL11B |  | ST3GAL4 |  | FAM71A | GLRX2 | LOC100128239 | ZSCAN25 |
| LOC100507066 | AQP3 |  | DKFZP434I0714 |  | LINC00311 | BCL2L10 | FGD5P1 | GGT1 |
| OR10J1 | FAM8A1 |  | LOC653653 |  | C17orf59 | NIT1 | HIRIP3 | PLA2G12B |
| S1PR4 | APBB1IP |  | LOC644961 |  | LOC100652791 | LBR | LINC00239 | HRH1 |
| DNLZ | EFNB2 |  | LOC100289511 |  | RUNX1-IT1 | IL21R-AS1 | TIGD6 | KLC3 |
| MESP2 | NDUFS8 |  | C16orf11 |  | TPPP2 | STBD1 | ATP10A | SMCR8 |
| BCL2A1 | USP22 |  | KLF5 |  | DRAP1 | CGB2 | OOEP | SMYD4 |
| AURKAIP1 | PRKAG3 |  | LOC730227 |  | GBA | STOML1 | GOLM1 | KIF21B |
| SNAI3 | GBA2 |  | ATAD3B |  | P2RY11 | C1RL | ITIH3 | USP36 |
| S100A2 | LOC100507410 |  | SPRN |  | HEPN1 | RCC1 | LOC643802 | PIN1 |
| FES | KLK4 |  | SLC4A3 |  | MDK | C9orf139 | GFI1 | UPK1A-AS1 |
| CIB3 | MAN2A2 |  | TNFRSF4 |  | FGL2 | IL23A | APOA1 | MMP27 |
| LOC401109 | UBXN1 |  | PFN1P2 |  | SLC16A11 | PDE6G | DHRS13 | DBNDD1 |
| HSFY1P1 | TGFBR3L |  | PPP2R5B |  | TMEM174 | APMAP | PRELP | C3orf80 |
| RPL11 | MC1R |  | NUDT8 |  | GLTPD1 | ARIH2OS | GHSR | CNPY3 |
| FOXD2-AS1 | SEC61A1 |  | ZNF154 |  | ARRDC1 | LGALS3 | MKNK2 |  |
| SLC52A3 | MANSC1 |  | LOC100130705 |  | LTB4R | NPPC | PDE4DIP |  |
| ZDHHC19 | TINAGL1 |  | C9orf40 |  | RAB24 | AKR7L | C2orf15 |  |
| EFNA1 | LGALS12 |  | BEGAIN |  | ABTB1 | SLC16A13 | SSRP1 |  |
| MRPS12 | LOC100128750 |  | ZNF252P-AS1 |  | TNNC2 | KCNK18 | ZSCAN22 |  |
| ZNF295-AS1 | FAM32A |  | FAM86B3P |  | CHCHD10 | BCL2L15 | HCG23 |  |
| IRF8 | FLYWCH2 |  | PLXNB2 |  | C3orf36 | PPP1R3D | ZC3H12A |  |
| TUBGCP6 | PRR14L |  | SUSD2 |  | B4GALT5 | RPL27 | LOC100506178 | |
| PGLYRP2 | ZNF669 |  | SPDYE3 |  | PABPC3 | LOC646626 | ZNRF3-AS1 | |
| TRIM46 | CCDC64B |  | GPSM1 |  | IL17C | RPL30 | CPNE2 |  |
| SIGLEC16 | ZBTB18 |  | MZT2B |  | GPR27 | SERPINB2 | FSCN1 |  |
| LOC728463 | ZNF551 |  | SNX22 |  | OR56A1 | C10orf128 | CABLES1 |  |
| CD300LF | HPD |  | CANT1 |  | VPS18 | ZNRF3 | MFAP2 |  |
| CABP4 | ITGAL |  | LOC148696 |  | TGFB1I1 | GPR4 | DHX15 |  |
| SRXN1 | CECR5-AS1 |  | NDUFA2 |  | MAGEF1 | ZNF513 | SSBP4 |  |
| MPDU1 |  |  | ZNF22 |  | LOC728752 | CEBPA | CYR61 |  |
| ZAR1 |  |  | CSH2 |  | DEFA4 | TMEM173 | FAM105A |  |
| APOF |  |  | PLSCR3 |  | TRAPPC2L | CLCNKB | TBC1D14 |  |
| CECR1 |  |  | CLCN2 |  | C16orf86 | CYP2D6 | FAM43A |  |
| THOC6 |  |  | POLD4 |  | EMILIN1 | KRT18 | PAOX |  |
| P2RY13 |  |  | ACP1 |  | RPS8 | FADS2 | CMAS |  |
| LTB4R2 |  |  | C8orf69 |  | C11orf42 | ANGPTL4 | ETS1 |  |
| NTHL1 |  |  | SLC29A4 |  | PP7080 | KRT12 | GLTSCR2 |  |
| ACTL10 |  |  | PPP4C |  | TRAPPC6A | ZNF770 | PIK3IP1 |  |
| SPRR2B |  |  | LOC100506100 |  | MED11 | LASP1 | NRN1 |  |
| BLOC1S4 |  |  | ZNF671 |  | KRTAP10-4 | CD207 | SIGIRR |  |
| LOC151174 |  |  | PSENEN |  | GHRL | TPBG | LDLRAD4 |  |
| FAM218A |  |  | LOC150776 |  | TCIRG1 | ZNF469 | PTGDS |  |
| GPR151 |  |  | HLA-DRB5 |  | FKBPL | LOC415056 | IL2RB |  |
| USP17L7 |  |  | SCNN1D |  | HBB | LOC731223 | IFI35 |  |
| RTP3 |  |  | KRT16 |  | CRKL | TMEM185B | PDK2 |  |
| C2orf62 |  |  | LOC100128682 |  | ZSCAN10 | FN3K | FCN3 |  |
| RPSAP9 |  |  | RHBDF1 |  | ZNF511 | MRPS26 | CDH16 |  |
| CXCL3 |  |  | FAM25A |  | C1S | PSMC5 | GIF |  |
| AIFM3 |  |  | SDCBP2-AS1 |  | KRTAP5-11 | LOC389641 | C3 |  |
| JAG2 |  |  | SAA1 |  | SLC16A3 | SLC45A4 | G0S2 |  |
